# Baicalein and Citric Acid Modulate Intestinal Morphology and Health Status in Laying Hens

**DOI:** 10.3390/vetsci12080706

**Published:** 2025-07-28

**Authors:** Yefei Zhou, Cunyi Qiu, Zhiding Zhou, Yanjie Zhang, Dunlin Zhang, Yao Cai, Jun Yuan, Shangxin Song, Zhihua Feng, Xinglong Wang

**Affiliations:** 1 Department of Food Science, Nanjing Xiaozhuang University, Nanjing 211171, China; 2 Gansu Polytechnic College of Animal Husbandry & Engineering, Wuwei 733006, China; 15797637570@163.com (C.Q.); zhihuaf@163.com (Z.F.); 3 College of Veterinary Medicine, Sichuan Agricultural University, Chengdu 611130, China; zhidingzhou@163.com; 4 College of Veterinary Medicine, Northwest A&F University, Yangling 712100, China; yjz2147@nwafu.edu.cn (Y.Z.); wxlong@nwsuaf.edu.cn (X.W.)

**Keywords:** layer, acidifier, baicalin, egg quality, intestinal flora

## Abstract

This study aimed to investigate the effects of baicalin and citric acid on egg production performance, egg quality, and the intestinal morphology and function of laying hens. A total of 600 Hy-Line Brown laying hens, 59 weeks old, were randomly allocated to four dietary treatments, with 10 replicates per treatment and 15 hens per replicate. The control group was fed a basal diet, while the other three groups were fed the basal diet supplemented with 150 mg/kg baicalin (B), 2000 mg/kg citric acid (CA), or 150 mg/kg baicalin plus 2000 mg/kg citric acid (B + CA), respectively. The experimental period lasted for 12 weeks, and the results indicated that neither the individual addition nor the combined application of baicalin and citric acid had a significant impact on the laying performance. However, compared with the control group, the baicalin and/or citric acid supplementation significantly increased the eggshell strength and Haugh unit. Additionally, the combination of baicalin and citric acid significantly increased the villus height and the villus height/crypt depth ratio in the duodenum and jejunum. It also enhanced the population of beneficial bacteria, such as *Lactobacillus* and *Bifidobacterium*, in the cecum and improved the activity of intestinal digestive enzymes, primarily disaccharidases. Furthermore, the addition of baicalin to the diet significantly increased the content of Secretory Immunoglobulin A in the ileum and jejunum after 12 weeks of feeding. These results suggest that the combination of baicalin and citric acid had a synergistic effect on the improvement of egg quality and intestinal morphology and function in laying hens. Overall, our findings provide important insights into the potential benefits of supplementing baicalin and citric acid in the diet of laying hens and may have practical implications for improving egg quality and poultry health status.

## Simple Summary

Baicalein exhibits various biological activities, including antibacterial, anti-inflammatory, and antioxidant properties. The positive regulatory effects of acidifiers on intestinal function are conducive to the absorption of Baicalein. This study aimed to investigate the effects of Baicalein and citric acid on laying performance, egg quality, and the intestine of laying hens. The research confirmed that the combination of citric acid and Baicalein has a synergistic effect in improving egg quality and enhancing intestinal morphology and function in laying hens.

## 1. Introduction

Antibiotics are widely used in large-scale and intensive poultry farming to enhance production efficiency and prevent or treat diseases. However, the excessive use of antibiotics in livestock and poultry feed has caused severe threats to the environment and human health. Prolonged and low-dose administration of antibiotics in feed can exert persistent selective pressure on resistant strains [1], leading to the emergence of novel resistant strains, which can infect animals and humans [2]. Research has demonstrated that the prohibition of ceftiofur injections in broiler chickens [3] significantly reduced the levels of cephalosporin-resistant *Salmonella* and *E. coli* in local chicken products and effectively reduced the incidence of cephalosporin-resistant *Salmonella* infection in humans. Therefore, it will be of significant practical importance to seek effective alternatives to antibiotics for livestock and poultry to mitigate the emergence of resistant strains and their potential risk of infecting humans.

Scutellaria, a traditional Chinese medicine and Chinese patent medicine, is the dried root of *Scutellaria baicalensis* Georgi, a member of the Lamiaceae family. Its main active ingredients include baicalin, wogonoside, Baicalein, and wogonin, which are extracted from Scutellaria [4]. Baicalin, a flavonoid compound, has been found to possess various pharmacological effects, such as heat-clearing and detoxifying properties, antibacterial and anti-inflammatory characteristics, and antioxidant properties. Its potential to reduce inflammatory responses and inhibit *Salmonella* infection in chicks has been demonstrated [5]. Moreover, the study by Li et al. [6] demonstrated that the supplementation of Scutellaria baicalensis extract in the diet could effectively mitigate the adverse effects induced by a Clostridium perfringens challenge by improving intestinal barrier function and tissue morphology and exerting a positive influence on the growth performance of the challenged chickens. In addition, Zhou et al. [7] reported that the inclusion of 200 mg/kg Baicalein in broiler diets significantly increased the total antioxidant capacity (T-AOC) activity, total superoxide dismutase (T-SOD), and glutathione peroxidase (GSH-Px) levels in the liver tissue. These findings suggest that Scutellaria could serve as a potential alternative to antibiotics in poultry feed; hence, it requires further studying.

Acidifiers are known to reduce pH in the intestine, thus enhancing the activity of proteolytic enzymes, promoting protein digestion, and inhibiting the proliferation of pathogenic bacteria in the gastrointestinal tract [8]. Owing to this potential as alternatives to antibiotics, acidifiers have been extensively studied and utilized in the feeds of various animals, including piglets, chickens, ducks, and sea bass [9,10,11]. Citric acid is one of the most commonly used organic acidifiers. Relevant studies have demonstrated that the supplementation of CA (particularly at a dosage of 10 g/kg of feed) in the diet of growing Japanese quails can enhance growth performance, immune response, and overall health [12]. In addition, Ahmed et al. [13] reported that the inclusion of 0.5% pure citric acid in the diet of weaned piglets significantly increased the lactic acid bacteria count in feces. Studies have indicated that the supplementation of 30 g/kg of CA increased the weight percentages of the proventriculus, gizzard, and ileum, as well as the villus length, crypt depth, and goblet cell count in the duodenum, jejunum, and ileum of broiler chickens. Furthermore, it enhanced the ileal crude protein (CP) digestibility, apparent metabolizable energy (AME), and total phosphorus (tP) [14].

Baicalein is a bioactive compound with antibacterial, anti-inflammatory, and antioxidant properties, representing a promising alternative to antibiotics [15]. However, its complex structure poses challenges for the complete absorption and utilization of its active components. Citric acid aids in regulating the intestinal microbiota, improving the intestinal environment, and enhancing digestive enzyme activity in chickens. Therefore, we hypothesize that the combined use of Baicalein and citric acid will be beneficial in augmenting the efficacy of Baicalein. In summary, this experiment aims to investigate the effects of Baicalein and citric acid on the laying performance, egg quality, intestinal morphology, and intestinal health status of laying hens.

## 2. Materials and Methods

### 2.1. Materials and Animals

Fifty-nine-week-old Hy-line brown commercial layers were provided by Nanjing Tianwei Farm (Nanjing, China). All the laying hens were housed in open houses with an average daily temperature of 30 to 36 °C. The Baicalein and citric acid, with purities of 98% and 99% respectively, were supplied by Qihe Huarui Animal Husbandry Co., Ltd. (Jinan, Shandong, China). Pepsin kits, trypsin activity detection kits, and amylase activity detection kits were purchased from the Nanjing Jiancheng Institute of Bioengineering. *Lactobacillus* and *Enterococcus* were provided by the Nanjing Xiaozhuang College Biology Teaching Experiment Central clinical separation preservation.

### 2.2. Experimental Animals and Diet

A total of 600 Hy-Line Brown laying hens, aged 59 weeks and with similar bodyweights, were randomly allocated to four dietary treatment groups, with 10 replicates per group. Each replicate consisted of 15 hens, which were housed in 5 cages (3 hens per cage). All the birds were adapted to the basal diet and environment for one week. The control group (CON) received a standard maize/soybean meal basal diet (Table 1) formulated according to the requirement of laying hens (National Research Council, 1994). For the other treatment groups, birds received a basal diet supplemented with 150 mg/kg of Baicalein (Baicalein), 2000 mg/kg of citric acid (CA), and 150 mg/kg of Baicalein plus 2000 mg/kg of citric acid (Baicalein + CA). The hens were housed in environmentally controlled three-tiered stepped wire cages (45 cm × 45 cm × 50 cm). The trial lasted for 12 weeks. All the hens were given ad libitum access to clean water with nipple drinkers and feed throughout the experiment. Hens were exposed to a photo-period cycle of 16 L:8 D.

### 2.3. Laying Performance

During the experimental period, eggs were collected daily at 09:00 h. Bird mortality was accurately recorded every day at 15:00. Total feed intake was recorded for each replicate at the end of the experiment. The laying feed conversion ratio (FCR) was computed as g feed consumption divided by g egg mass (g feed/g egg mass). For laying performance, the daily egg production and egg weight per cage were recorded, and the average values for each hen within the replicate were calculated.

### 2.4. Egg Quality Measurements

Egg quality indices were determined every week by randomly collecting 10 eggs per treatment. Eggshell-breaking strength was measured using an Egg Force Reader (Orka Technology Ltd., Ramat Hasharon, Israel). Eggshell thickness was measured using a Peacock dial gauge (P-1 Model, Meg Co., Ltd. Ozaki, Japan) after removing the shell membrane, representing the average thickness of the upper, middle, and lower end of the shell. Haugh units (HUs), albumin height, and yolk color were analyzed using an Egg Multi Tester EMT-5200 (Robotmation Co., Ltd. Tokyo, Japan). Egg length (L) and width (W) were measured using Vernier calipers with the least count of 0.01 mm. The egg shape index (SI) was determined from the egg length and width.

### 2.5. Slaughtering and Sampling

At 68 and 72 weeks of age, one laying hen, with a bodyweight close to the average of each replicate cage, was euthanized per replicate. The duodenum, jejunum, ileum, and cecum were then collected. The duodenum and jejunum were longitudinally incised, and the intestinal mucosa was scraped using sterile slides to detect the secretion of digestive enzymes and secretory sIgA Cecal contents were collected into sterile microtubes for bacterial enumeration. Subsequently, samples of the intestinal mucosa and cecal contents were placed in liquid nitrogen and then transferred to a −80 °C freezer for storage.

### 2.6. Intestinal Morphology

Paraffin sections of small intestinal tissue were prepared, and for each section, 10 fields of view were selected. The intestinal villus height (VH) and crypt depth (CD) were observed and measured using an optical microscope, and the villus height-to-crypt depth ratio (VH/CD) was calculated. Additionally, the intestinal wall thickness (WT) was measured, encompassing the mucosa, submucosa, muscularis propria, and serosa layers.

### 2.7. Secretory Immunoglobulin A and Digestive Enzyme Analyses

After intestinal mucosa had been defrosted, homogenized, and centrifuged, the **sIgA** levels were determined using ELISA (Shanghai Fanyin Biotechnology Co., Ltd., Shanghai, China). The protein content of the intestinal mucosa was determined using the Bradford method. For the remaining samples to be tested, enzyme activities of maltase, invertase, amylase, trypsin, and lipase were measured following the detection method provided in the commercial kits, after appropriate sample processing. Each sample was analyzed in triplicate wells (with an intra-batch coefficient of variation ≤ 5% and an inter-batch coefficient of variation ≤ 10% for the kits).

### 2.8. Detection of Intestinal Bacteria

Ten chickens were randomly selected from each group, and 2 g of cecal contents were taken. *Lactobacillus*, *Bifidobacterium*, and *Escherichia coli* were isolated and counted. The microbial enumerations of total aerobes, *Escherichia coli*, *Lactobacillus,* and *Bifidobacteria* were plated using selective agar media [16]. The cecal samples were serially diluted with sterile physiological saline according to the procedure described by Yang et al. [17]. Total aerobes and *E. coli* were counted on trypticase soy agar and eosin-methylene blue agars, respectively, and incubated aerobically at 37 °C for 24 h. *Lactobacillus* and *Bifidobacteria* were cultivated on a selective medium for lactic acid bacteria and BBL agar medium, respectively. Anaerobic incubation was achieved under an anaerobic atmosphere (80% N_2_, 15% CO_2_, and 5% H_2_) at 37 °C for 48 h without agitation. The result was lg (CFU/g), that is, the total number of colonies per gram of cecal contents.

### 2.9. Data Processing and Analysis

All data were subjected to a 2 × 2 factorial analysis of variance (ANOVA) using SPSS 20.0 (SPSS Inc., Chicago, IL, USA) statistical software. Initially, the Shapiro–Wilk (S-W) test was employed to assess the normality of the data within each group. The ANOVA was conducted using the General Linear Model (GLM) procedure, and Bonferroni multiple comparison tests were utilized to determine significant differences among different dietary treatments. A statistically significant difference was defined as *p* < 0.05.

## 3. Results and Analysis

### 3.1. Laying Performance

Table 2 shows that there were no significant effects (*p* > 0.05) on the laying rate, average daily feed intake, feed-to-egg ratio, and egg quality of laying hens fed diets containing baicalin or citric acid compared to those of the control group.

### 3.2. Egg Quality Measurements

The results of Table 3 demonstrate that the addition of Baicalein or citric acid alone to the diet did not result in significant changes in egg quality indicators, such as the egg shape index, shell strength, shell thickness, yolk height, and Haugh unit (*p* > 0.05) compared to the control group. However, the combined use of Baicalein and citric acid significantly improved the shell strength and Haugh unit.

### 3.3. Intestinal Morphology

As shown in Table 4, after 8 weeks of feeding with a basal diet supplemented with baicalin and citric acid, the effects on the intestinal morphology of laying hens were mainly concentrated in the duodenum and ileum. Specifically, compared with the basal diet group, the combined use of baicalin and citric acid could significantly increase the villus height and the VH:CD ratio in the duodenum, as well as the VH:CD ratio in the ileum. In addition, the sole addition of baicalin also demonstrated an increasing effect on the VH:CD ratio of the duodenum. Overall, the combined application of baicalin and citric acid in the diet enhanced the absorptive function of the duodenum and jejunum. After 12 weeks of feeding with a basal diet supplemented with baicalin and citric acid, the effects on the intestinal morphology of laying hens were primarily focused on the jejunum. Compared with the basal diet group, both the sole addition of baicalin and the combined addition of baicalin and citric acid significantly increased the thickness of the jejunal wall. However, the sole addition of citric acid had no significant impact on the small intestinal morphology.

### 3.4. Secretory Immunoglobulin A and Digestive Enzyme Analyses

Based on the results presented in Table 5, supplementation of baicalin in the diet of laying hens for eight weeks significantly increased the content of sIgA in the ileum. Similarly, after 12 weeks of baicalin supplementation, the content of sIgA in both the duodenum and ileum of the laying hens significantly increased. Moreover, citric acid plus baicalin supplementation significantly enhanced the sIgA content in the duodenum and ileum of laying hens. However, the use of citric acid alone did not significantly affect the sIgA content in the intestinal segments of laying hens (*p* > 0.05).

As shown in Table 6, the supplementation of baicalin alone or baicalin plus citric acid for eight weeks significantly enhanced the activities of maltase and lactase in the duodenum of laying hens. Moreover, the addition of citric acid alone to the diet significantly increased maltase activity. After 12 weeks of dietary supplementation with baicalin or citric acid, lipase activity in the jejunum of laying hens significantly increased. Furthermore, the concurrent administration of baicalin and citric acid had a remarkable synergistic effect, leading to a more significant increase in lipase activity than the individual addition of these components.

### 3.5. Intestinal Microbiome Detection

Based on the results presented in Table 7, it was observed that the addition of citric acid alone or baicalin plus citric acid in the diet resulted in a significant increase in the population of *lactobacilli* and *bifidobacteria* in the cecum of laying hens after 8/12 weeks of treatment. However, the addition of baicalin alone to the diet resulted in a significant increase in the population of *bifidobacteria* in the cecum of laying hens only after 12 weeks of treatment.

## 4. Discussion

Against the backdrop of reducing antibacterial drugs in the current layer farming industry, it is widely accepted that acidifiers can effectively enhance feed utilization, production performance, and health in laying hens. Specifically, exogenous H^+^ provided by acidifiers can lower the pH value of food in the stomach and promote the activity of gastric proteases. Additionally, acidifiers can increase the activity of Na^+^/K^+^-ATPase, which directly participates in the digestion and absorption processes of poultry [18]. Research has demonstrated that including 0.6% citric acid in poultry diets significantly improves egg production and reduces the feed–egg ratio in Japanese quails [19]. Another study in layers at 44 weeks of age reported no beneficial influence of dietary acidifiers on productive performance [20]. In this study, the addition of 2000 mg/kg (0.2%) CA to the basal diet had no significant effects on the production performance and egg quality of laying hens. That might have been caused by the breed of the hens and an inadequate supplementation level of citric acid. However, the reason for this will need to be further studied and confirmed. Flavonoid compounds can indirectly promote the synthesis of egg yolk precursors and follicular development in laying hens via estrogen (E_2_), thereby enhancing their production performance [21,22]. Research by An et al. [23] showed that the addition of 5 g/kg of *S. baicalensis* extracts to a poultry diet effectively increased the egg weight. Dai et al. [24] also reported that the addition of hawthorn flavonoids to a poultry diet increased the egg production rate of laying hens. In the present study, dietary supplementation of baicalin did not significantly affect egg production, average daily feed intake, feed conversion ratio, or egg quality of poultry compared to those of the control group. This result is consistent with the findings of [25], whose study showed that citrus flavonoid supplementation in poultry diet had no significant effect on egg production performance and egg quality (*p* > 0.05). To determine the reasons for the observed differences, in-depth research on the chemical structural variations among different flavonoid compounds and their impacts on the reproductive system of laying hens [26] is necessary. In terms of egg quality, relevant studies have demonstrated that flavonoids can regulate calcium metabolism through their estrogen-like effects to enhance eggshell thickness [27]. For instance, dietary supplementation with moringa leaf flavonoids (MFM) has been shown to increase the eggshell strength of duck eggs [28]. In the present study, although the individual addition of citric acid or Baicalein to the diet had no significant impact on egg quality, the combined use of citric acid and Baicalein significantly improved the eggshell strength and Haugh unit of eggs. This outcome may be attributed to citric acid increasing calcium solubility by lowering the intestinal pH, thereby enhancing the estrogen-like effects of Baicalein (a flavonoid) and regulating calcium metabolism and absorption [29,30].

The small intestine is the leading site for food digestion and absorption, which strictly depends on the integrity of the small intestine structure. A study showed that baicalin can improve the intestinal structure of mice after Ionizing Radiation (IR), the villus height, and crypt number of the small intestine of a mouse [31]. As crucial indicators for the intestinal health of animals, villus height and crypt depth directly affect the absorption capacity of the intestinal mucosa. The addition of flavonoid compounds such as luteolin and naringin, which are similar to baicalin, to broiler diets, significantly increased the villus height and VH:CD ratio of the duodenum and jejunum [32]. In the current study, the addition of baicalin to the diet significantly increased the VH:CD ratio of the duodenum and ileum in laying hens. The above results may be because baicalin enhanced the proliferation and differentiation ability of intestinal stem cells.

After the activation of mucosal immunity in the intestinal tract, locally produced antibodies in the intestinal mucosa appeared earlier than serum antibodies, with higher titers and longer duration, and mucosal immunity is one of the most important barriers for the body to resist pathogen infection and other potentially deleterious microorganisms [33]. In avian species, the sIgA is regarded as the main immune barrier that maintains the homeostasis of the symbiotic flora [34]. Dietary supplementation has been tied to improvements in humoral immunity among laying hens [35]. In this study, the influence of baicalin on the mucosal immunity of laying hens was assessed by measuring the level of sIgA in the intestinal mucosa. Baicalin-supplemented diets fed to laying hens significantly elevated the sIgA level in the duodenum and jejunum compared to that of the control group, indicating that baicalin is beneficial for enhancing the intestinal mucosal immunity in laying hens. Previous studies showed that the addition of flavonoids to diets is beneficial for increasing antibody titers in broilers [7,36,37], demonstrating the immunomodulatory effects of flavonoids. Interestingly, although citric acid alone had no significant effect on the level of sIgA in different segments of the intestine in laying hens, it seemed to synergistically affect the immunomodulatory effect of baicalin. This may be because the acidifying agent improved the intestinal environment and increased the bioavailability of baicalin [38,39].

As for poultry, the increase in the content and activity of digestive enzymes, such as maltase, amylase, and lipase, is conducive to enhancing the absorption capacity of nutrients through the intestinal tract. The study conducted by Pereira et al. [40] showed that flavonoids regulate glucose absorption by inhibiting sucrase activity. They showed that a rutin concentration of 3.125–400 μM reduced the maltase activity in the rat duodenum but had no significant effect on lactase activity, and kaempferitrin significantly reduced the specific activities of maltase and sucrase in the rat duodenum. However, in this study, we obtained the opposite results. After feeding the laying hens with diets supplemented with baicalin alone or baicalin plus citric acid for eight weeks, the maltase and lactase activities in the duodenum of laying hens significantly increased (*p* < 0.05). We speculate that different flavonoids may have different target sites in regulating glucose homeostasis; hence, there is a discrepancy in the results obtained.

The pH value plays an important regulatory role in the balance of intestinal microbiota [41]. Due to the increased demand for calcium in laying hens, a large amount of calcium carbonate powder is often added to the feed, which could lead to excessive consumption of acidic substances in the intestine. Therefore, the addition of organic acidifiers is beneficial for maintaining an appropriate low-pH environment in the intestine, which is conducive to the digestive and absorptive functions of the intestine and the growth of beneficial bacteria, because beneficial bacteria usually tolerate low pH values [42]. Studies have shown that dietary acidophilic lactobacilli enhanced the barrier function of the intestinal mucosa in birds [43], and Chichlowski et al. [41] demonstrated that Lactobacillus increased the height of villi and decreased the depth of crypts in the ceca of chicks, thereby promoting intestinal function. In the present study, the addition of citric acid to the diet significantly increased the content of *Lactobacillus* and *Bifidobacterium* in the cecum, corroborating the findings of Wang et al. [44]. The presence of *Lactobacillus* can be increased by the addition of benzoic acid (BA) and alpha-amylase (AL) to the diet. Although there is evidence that flavonoids could upregulate the production of beneficial bacteria, stimulate the secretion of secretory immunoglobulin, prevent the adhesion of pathogens and toxins, and maintain the balance of gut microbiota through the effect of occupation and secretion of antibacterial peptides [45], in this study, except for the observed increase in the number of *Bifidobacterium* in the cecum after 12 weeks of adding baicalin to the diet, baicalin did not affect the number of other cecal probiotics at other stages. Intestinal probiotics such as *Bifidobacterium* and *Lactobacillus* are critical in the metabolism of flavonoids because they increase the bioactivity and bioavailability of flavonoids through reduction, decarboxylation, and demethylation reactions [46]. Furthermore, probiotics typically exhibit high tolerance to low pH, and dietary citric acid can enhance the vitality of probiotic populations by providing an appropriate low-pH environment in the intestine, thus improving the bioavailability of flavonoids [47].

## 5. Conclusions

In conclusion, this study shows that dietary supplementation with Baicalein and/or citric acid increases egg quality, intestinal morphology, immune status, the activity of intestinal digestive enzymes, and the population of beneficial bacteria. These findings contribute to a deeper understanding of the potential mechanisms by which the combined use of Baicalein and/or citric acid may improve egg quality and host health status in laying hens.

## Figures and Tables

**Table 1:** Basal diet composition and nutrient level of laying hens.

Items	Content	Items	Content
Diet Composition (% of total diet composition)		Nutrient levels ^2^	
Corn/%	59.00	Metabolizable Energy/(MJ/kg)	11.12
Soybean Meal/%	23.00	Crude Protein/%	15.98
Wheat bran/%	1.50	Calcium/%	3.67
Soybean oil/%	0.50	Total Phosphorus/%	0.61
Dicalcium Phosphate/%	1.50	Crude Fiber/%	3.90
Fish Meal/%	1.30	Crude Ash/%	2.35
Limestone/%	8.00	Lysine/%	0.85
Salt/%	0.50	Methionine/%	0.28
Methionine/%	0.50	(Methionine + Cystine)/%	0.52
Lysine/%	0.20		
Premix/% ^1^	4.00		

^1^ Note: Premixes are provided per kg of the diet, Vitamin A 4000 IU, Vitamin D_3_ 1200 IU, Vitamin E_6_ mg, Vitamin B_1_ 1.4 mg, Vitamin B_2_ 3 mg, Vitamin B_6_ 1.0 mg, Vitamin B_12_ 0.01 mg, Pantothenic Acid 7.5 mg, Choline 500 mg, Biotin 0.15 mg, Ca 7500 mg, P 3000 mg, Mn (MnSO_4_.H_2_O) 72 mg, Zn (ZnO) 56 mg, Fe (FeSO_4_.7H_2_O) 60 mg, Cu (CuSO_4_.5H_2_O) 8 mg, I (Ca (IO_3_) _2_.H_2_O) 0.5 mg, Se 0.1 mg. ^2^ Crude protein, calcium, phosphorus, crude fibre and crude ash were a measured value and the others were calculated values.

**Table 2:** Effects of Baicalein and citric acid on the performance of laying hens.

Items	CON	Baicalein	CA	Baicalein + CA	SEM	*p* Value
Egg production, %	84.06	84.62	84.43	84.91	1.65	0.19
Egg mass, g/day	52.48	53.20	53.76	54.26	2.06	0.97
Average Daily Feed Intake, g/d/bird	104.15	105.26	104.95	105.75	0.78	0.82
FCR, g/g	2.12	2.16	2.23	2.09	0.15	0.20

**Table 3:** Effects of Baicalein and citric acid on egg quality of laying hens.

Item	CON	Baicalein	CA	Baicalein + CA	SEM ^1^	*p* Value
Egg-Shaped Index	78.82	79.12	78.63	79.79	1.02	0.28
Eggshell strength, kg/cm^2^	3.02 ^b^	3.54 ^b^	3.45 ^b^	3.67 ^a^	0.03	0.01
Eggshell thickness, mm	0.47	0.43	0.47	0.48	0.14	0.15
Yolk height, mm	6.37	6.47	6.43	6.49	0.16	0.19
Haugh Unit	76.58 ^b^	77.36 ^b^	78.62 ^b^	79.65 ^a^	0.02	0.03

^1^ SEM, standard error of mean. Each mean represents 10 individual eggs. ^a,b^ Means values with different superscript letters in the same row indicate significant differences (*p* < 0.05).

**Table 4:** Effects of Baicalein and citric acid on the intestinal morphology of laying hens.

Items ^1^		CON	Baicalein	CA	Baicalein + CA	SEM ^2^	*p* Value
Week 68							
Duodenum	VH, μm	1038.83 ^b^	1047.14 ^b^	1076.22 ^b^	1107.36 ^a^	21.20	0.01
	CD, μm	170.77	136.60	174.74	145.77	1.53	0.92
	VH:CD ratio	6.17 ^b^	7.80 ^a^	6.16 ^b^	7.77 ^a^	0.63	0.02
	WT, μm	125.77	126.49	126.09	127.28	4.30	0.87
Jejunum	VH, μm	822.30	983.20	927.54	992.81	16.54	0.48
	CD, μm	113.05	178.30	122.12	148.43	1.67	0.91
	VH:CD ratio	7.39	5.65	8.09	6.76	0.39	0.39
	WT, μm	110.24	120.19	119.37	126.49	5.45	0.62
Ileum	VH, μm	497.19	606.94	511.58	654.71	11.82	0.71
	CD, μm	101.90	129.01	111.07	126.30	2.12	0.38
	VH:CD ratio	4.88 ^b^	4.64 ^b^	4.54 ^b^	5.06 ^a^	0.03	0.03
	WT, μm	132.27	129.54	129.43	141.09	7.17	0.42
Week 72							
Duodenum	VH, μm	1207.82	1210.84	1261.93	1269.10	18.14	0.62
	CD, μm	222.85	180.77	196.49	182.85	1.12	0.52
	VH:CD ratio	5.15	6.67	6.19	6.68	0.34	0.25
	WT, μm	112.33	123.07	122.86	127.92	2.73	0.35
Jejunum	VH, μm	632.70	848.20	746.65	790.78	14.49	0.32
	CD, μm	139.79	154.01	132.87	122.08	1.34	0.76
	VH:CD ratio	4.28	5.19	5.41	6.40	0.56	0.32
	WT, μm	107.33 ^b^	129.15 ^a^	106.65 ^b^	128.27 ^a^	1.23	0.01
Ileum	VH, μm	620.79	586.25	623.74	629.06	13.02	0.32
	CD, μm	110.10	102.32	104.18	98.02	1.59	0.43
	VH:CD ratio	5.38	5.21	5.55	6.04	0.46	0.23
	WT, μm	110.73	121.82	112.94	122.36	2.13	0.54

^1^ VH, villus height; CD, crypt depth; VH:CD ratio, villus height to crypt depth ratio and WT, intestinal wall thickness. ^2^ SEM, standard error of mean. Each mean represents 10 individual birds. ^a,b^ Means values with different superscript letters in the same row indicate significant differences (*p* < 0.05).

**Table 5:** Effects of Baicalein and citric Acid on sIgA (ng/100 mg intestinal mucosa scrapings) in the intestinal mucosa of laying hens.

Item	CON	Baicalein	CA	Baicalein + CA	SEM ^1^	*p* Value
Week68						
Duodenum	7.55	7.44	7.78	7.99	0.32	0.81
Jejunum	7.14	7.02	7.17	7.22	0.12	0.31
Ileum	6.87 ^b^	7.83 ^a^	6.78 ^b^	8.28 ^a^	0.28	0.01
Week72						
Duodenum	7.18	7.09	7.37	7.57	0.29	0.24
Jejunum	5.42 ^b^	6.89 ^a^	5.39 ^b^	7.08 ^a^	0.26	0.02
Ileum	6.95 ^b^	8.28 ^a^	6.92 ^b^	8.19 ^a^	0.13	0.01

^1^ SEM, standard error of mean. Each mean represents 10 individual birds. ^a,b^ Means values with different superscript letters in the same row indicate significant differences (*p* < 0.05).

**Table 6:** Effects of Baicalein and citric acid on intestinal digestive enzymes in the intestinal mucosa of laying hens.

Item		CON	Baicalein	CA	Baicalein + CA	SEM ^1^	*p* Value
Week 68							
Duodenum	Maltase, U/mg	314.77	333.84	313.84	385.66	3.76	0.13
	Sucrase, U/mg	21.57	24.87	23.51	26.01	1.14	0.10
	Amylase, U/mg	125.62	139.28	131.19	147.06	6.13	0.77
	Trypsin, U/mg	702.37	741.69	721.71	769.91	29.18	0.81
	Lipase, U/g	21.18	23.65	24.53	35.45	1.18	0.34
Jejunum	Maltase, U/mg	478.43 ^c^	621.72 ^a^	560.9 ^b^	715.68 ^a^	2.35	0.01
	Sucrase, U/mg	52.42 ^b^	62.13 ^a^	58.34 ^b^	67.83 ^a^	3.14	0.05
	Amylase, U/mg	31.43	32.46	30.89	34.78	2.63	0.40
	Trypsin, U/mg	309.06	347.81	328.56	406.07	4.95	0.15
	Lipase, U/g	98.87	113.46	108.51	119.83	1.14	0.31
Ileum	Maltase, U/mg	457.81	505.89	460.92	520.13	13.08	1.37
	Sucrase, U/mg	34.12	37.26	35.39	40.97	2.18	0.22
	Amylase, U/mg	19.37	22.32	20.56	24.51	1.19	0.26
	Trypsin, U/mg	270.36	365.02	306.83	386.75	11.52	0.29
	Lipase, U/g	61.79	72.69	77.08	91.70	1.29	0.11
Week 72							
Duodenum	Maltase, U/mg	304.29	341.73	309.92	367.82	4.17	0.83
	Sucrase, U/mg	30.24	37.03	33.86	38.75	5.28	0.29
	Amylase, U/mg	123.49	131.86	129.83	146.16	3.78	0.59
	Trypsin, U/mg	704.84	741.38	723.19	768.38	9.88	0.84
	Lipase, U/g	20.77 ^c^	28.23 ^b^	27.90 ^b^	39.81 ^a^	2.02	0.04
Jejunum	Maltase, U/mg	469.06	634.19	590.84	685.79	11.89	0.40
	Sucrase, U/mg	34.89	39.54	40.29	49.36	8.23	0.14
	Amylase, U/mg	106.82	112.39	110.88	124.69	4.36	0.54
	Trypsin, U/mg	726.91	788.17	737.92	826.05	28.17	0.50
	Lipase, U/g	23.50	26.08	26.65	33.16	2.38	0.60
Ileum	Maltase, U/mg	470.31	519.06	490.48	537.81	9.63	0.93
	Sucrase, U/mg	30.86	35.15	32.97	39.81	3.52	0.14
	Amylase, U/mg	121.79	137.81	126.90	146.03	5.26	0.31
	Trypsin, U/mg	774.29	831.03	818.69	845.62	31.76	0.48
	Lipase, U/g	28.24	30.77	29.79	36.49	1.22	0.21

^1^ SEM, standard error of mean. Each mean represents 10 individual birds. ^a,b,c^ Means values with different superscript letters in the same row indicate significant differences (*p* < 0.05).

**Table 7:** Effects of Baicalein and citric acid on microbial count (lg CFU/g) in the cecal digesta of laying hens.

Item	CON	Baicalein	CA	Baicalein + CA	SEM ^1^	*p* Value
Week 68						
Total aerobes	7.55	7.44	7.78	7.99	0.32	0.81
Lactobacilli	7.87 ^b^	7.14 ^b^	8.02 ^a^	8.22 ^a^	0.12	0.02
*Bifidobacteria*	6.87 ^b^	7.78 ^b^	8.28 ^a^	8.85 ^a^	0.11	0.01
*E. coli*	6.18	6.08	6.21	5.90	0.07	0.33
Week 72						
Total aerobes	7.43	7.49	6.68	6.70	0.40	0.32
Lactobacilli	8.63 ^b^	9.84 ^b^	10.95 ^a^	11.93 ^a^	1.07	0.01
*Bifidobacteria*	7.01 ^b^	9.83 ^a^	9.49 ^a^	10.79 ^a^	0.17	0.03
*E. coli*	6.20	7.83	8.19	9.28	0.16	0.31

^1^ SEM, standard error of mean. Each mean represents 10 individual birds. ^a,b^ Means values with different superscript letters in the same row indicate significant differences (*p* < 0.05).

## Data Availability

All relevant data are within the paper.

## Abbreviations

The following abbreviations are used in this manuscript:

FCR	feed conversion ratio
CA	citric acid
CON	control group
VH	villus height
CD	crypt depth
WT	wall thickness
V/C	villus height/crypt depth
sIgA	Secretory Immunoglobulin A
